# Using Magnetic Molecularly Imprinted Polymer Technology for Determination of Fish Serum Glucose Levels

**DOI:** 10.3390/polym16111538

**Published:** 2024-05-29

**Authors:** Boxuan Yao, Long Gu, Li Huang, Ruichun Li, Ze Fan, Zhongxiang Chen, Dongli Qin, Lei Gao

**Affiliations:** 1 Heilongjiang River Fisheries Research Institute, Chinese Academy of Fishery Sciences, Harbin 150070, China; yaobox2626@163.com (B.Y.); gulong0320@163.com (L.G.); huangli@hrfri.ac.cn (L.H.); 13403557425@163.com (R.L.); fanze@hrfri.ac.cn (Z.F.); chenzhongxiang@hrfri.ac.cn (Z.C.); 2 College of Food Science and Engineering, Dalian Ocean University, Dalian 116023, China; 3 Supervision, Inspection and Testing Center for Fishery Environment and Aquatic Products (Harbin), Ministry of Agriculture and Rural Affairs, Harbin 150070, China; 4 Key Laboratory of Control of Quality and Safety for Aquatic Products, Ministry of Agriculture and Rural Affairs, Beijing 100141, China

**Keywords:** glucose, serum, magnetic solid-phase extraction, selective extraction, derivatization

## Abstract

In this study, a highly efficient magnetic molecularly imprinted polymer nanocomposite material was prepared using multi-walled carbon nanotubes as carriers. The characterization of the obtained nanocomposite material was conducted using Fourier transform infrared spectroscopy, a vibrating sample magnetometer, a thermogravimetric analyzer, a scanning electron microscope, and a transmission electron microscope. The adsorption properties of the nanocomposite material were evaluated through adsorption experiments, including static adsorption, dynamic adsorption, and selective recognition studies. The prepared nanocomposite material, serving as a selective adsorbent, was applied in magnetic solid-phase extraction. Subsequently, the derivatized samples were analyzed for glucose in fish serum using liquid chromatography–tandem mass spectrometry. Under optimal conditions, the detection limit was 0.30 ng/mL, the quantitation limit was 0.99 ng/mL, satisfactory spiked recovery rates were obtained, and the relative standard deviation was less than 1.1%. Using 2-deoxy-D-ribose as the template molecule and a structural analog of glucose allowed us to eliminate the potential template leakage in qualitative and quantitative analyses, effectively avoiding the issues of false positives and potential quantitative errors, compared to traditional methods. A method for detecting glucose levels in fish serum based on molecularly imprinted polymer technology has been successfully developed to determine the stress and health levels of fish.

## 1. Introduction

Glucose is one of the most widely distributed and important monosaccharides in nature. Because of the nutritional value of glucose, it has a broad application prospect in the food industry, and it is the main energy source for biological activities, as well as a common nutrient [1]. In the aquaculture field, the glucose level in fish blood has a crucial impact on the nutritional status and stress response of fish. The nutritional status and stress level of fish can be assessed through their blood glucose levels, hence accurate detection of the glucose content in fish blood is important [2]. Currently, common methods for determining monosaccharides such as glucose in food include the high-performance liquid chromatography–evaporative light scattering detection (HPLC-ELSD) method [3,4,5], the high-performance liquid chromatography–refractive index detection (HPLC-RID) method [6,7,8], the biosensor method [9], the nanowire aerogel method [10], and the glucose assay kit method [11]. Among them, the HPLC-ELSD method is classical and widely used for glucose detection, with well-developed procedures. However, the existing methods have some shortcomings, such as insufficient limits of detection (LOD) and quantitation (LOQ), low sensitivity when dealing with complex matrices, and difficulty in qualitative analysis, which may not meet the experimental requirements. High-performance liquid chromatography–tandem mass spectrometry (HPLC-MS/MS) is widely used due to its high sensitivity. However, glucose molecules cannot carry electrons on the electrospray ionization (ESI) source and cannot be detected directly on HPLC-MS/MS. To detect glucose on HPLC-MS/MS, the derivatization of glucose molecules is required [12]. This method offers a higher sensitivity and a simpler experimental operation, addressing the low sensitivity issue encountered in glucose instrumental analysis.

Molecularly imprinted polymers (MIPs) are polymer materials with binding sites. MIPs have garnered significant attention due to their benefits of straightforward preparation and outstanding physical and chemical characteristics. MIPs are intricately cross-linked porous polymers featuring imprinted voids tailored to match the shape and dimensions of the template molecule. The functional moieties within MIPs exhibit selective binding to the desired molecule through mechanisms such as hydrogen bonding, hydrophobic interactions, and electrostatic forces [13]. They are widely used for pretreatment and selective enrichment of target compounds in complex matrices [14,15,16]. MIPs exhibit extremely high selectivity towards target molecules and are extensively employed in solid-phase extraction (SPE) [17], serving as suitable alternatives to solid adsorbents. The adsorption capacity and selectivity of the adsorbent are crucial factors during the purification of analytes by SPE. Traditional adsorbents often possess low adsorption capacity and insufficient selectivity. Therefore, the preparation of adsorption materials with high adsorption capacity and high selectivity is of great significance for improving SPE. Compared to classical solid-phase adsorbents, MIPs offer many advantages, yet they still have several issues to address. According to the literature, MIPs using activated silica gel (SiO_2_@NH_2_) particles as carrier particles with glucose as the template molecule [18] have two main drawbacks, including insufficient desorption of the template molecules, resulting in low adsorption of glucose and, more importantly, false positive detection due to the incomplete elution of the template molecules. To address the false positive issue caused by the similarity between the template molecule and the analyte, the use of special MIPs can be effective. MIPs utilize compound structures similar to the analytical target as template molecules. During the preparation process, residual template molecules are different from the target substance, thereby avoiding false positive detection. Conventional MIPs materials typically use SiO_2_@NH_2_ as the carrier for adsorbents. However, factors such as the aggregation of nanomaterials may lead to a low adsorption capacity. This might be due to the inappropriate selection of the adsorption carrier. Carbon nanotubes (CNTs) possess high chemical stability and a large surface area, making them excellent carriers for improving adsorption performance [19]. In this study, CNTs were used as the carrier for the adsorbents.

Typically, sample preparation related to fish involves SPE, yet the drawback of the inadequate detection rates of SPE becomes magnified when dealing with large sample quantities. To address this issue, magnetic solid-phase extraction (MSPE) can effectively shorten the enrichment operation time. During the MSPE process, magnetic adsorbents are dispersed directly in the extract to adsorb analytes. If the adsorbent is selective, the matrix interference can be reduced by using MIPs, increasing sensitivity to the target substance. After the extraction process, the magnetic adsorbents in the extract are rapidly collected using an external magnet [20,21]. Carbon nanotubes are used as carriers, with magnetic Fe_3_O_4_ as the core and 2-deoxy-D-ribose as the template molecule. To synthesize magnetic molecularly imprinted polymers (MMIPs), we chose 2-deoxy-D-ribose as the template molecule because its structural formula is very similar to that of glucose molecules. The spatial geometries in the modified surface of the polymer facilitated the adsorption of the glucose molecules by the material.

By avoiding centrifugation with an external magnetic field, complex separation processes are bypassed, enabling the rapid separation from composite matrices and the development of a highly selective and reusable analytical procedure [22]. After treating fish serum with MSPE using MMIPs, this study derivatizes the eluted glucose with 1-phenyl-3-methyl-5-pyrazolone (PMP). Under alkaline conditions, the reducing end of one glucose molecule forms a stable derivative chromophore with two PMP molecules, enabling qualitative and quantitative analyses using a diode array detector (DAD) [23,24]. Furthermore, these derivatives are electron-carrying, exhibiting signals on mass spectrometry (MS), thereby enhancing the analysis signal of monosaccharide–2PMP derivatives in MS. By increasing the sensitivity to glucose detection through derivatization, coupled with magnetic dynamic separation, the avoidance of false positives through special template molecules, the high selectivity of MIPs technology, and the high sensitivity of HPLC-MS/MS, the inherent shortcomings of current methods for detecting the glucose content in fish serum can be effectively addressed.

In this study, MMIPs were synthesized using magnetic carbon nanotubes (MCNTs) as the adsorption carrier, 2-deoxy-D-ribose as the template molecule, and 3-aminophenylboronic acid as the functional monomer. The adsorption mechanism of MMIPs was explored through characterization. Additionally, experiments such as adsorption modeling were conducted to study the adsorption mechanism and evaluate the selective recognition capability of MMIPs, along with optimizing the elution conditions. After material synthesis, MMIPs were used as adsorbents for the MSPE treatment of the fish serum samples. Subsequently, the eluted monosaccharide molecules were derivatized with PMP and qualitatively and quantitatively analyzed using HPLC-MS/MS.

## 2. Materials and Methods

### 2.1. Materials and Reagents

Mass grade acetonitrile (ACN), methanol, and formic acid were purchased from Fisher Scientific (Waltham, MA, USA). Carbon nanotubes were obtained from Nanotech Port (Shenzhen, China). Standard monosaccharide and disaccharide (glucose, sucrose) solutions, as well as PMP, were acquired from Aladdin (Shanghai, China). Sodium acetate, FeCl_3_·6H_2_O, ethylene glycol, isopropanol, ammonia solution, ammonium persulfate, and tetramethyl orthosilicate were obtained from Anpel (Shanghai, China). 3-aminophenylboronic acid and 2-deoxy-D-ribose were purchased from Yuanye Bio-Technology Co (Shanghai, China). N,N’-methylenebisacrylamide was obtained from RHAWN (Shanghai, China). MilliQ system (Merck Millipore, Billerica, MA, USA) was used for water purification. Chromatographic separation was performed using an ACQUITY BEH C18 column (1 × 50 mm, particle size 1.7 μm). The PMP-methanol solution (87 mg/mL) was stored in a light-sealed environment at 2–8 °C.

### 2.2. Standard Solutions

Stock solutions of glucose and sucrose with a concentration of 20 mg/mL were prepared in deionized water and stored at 4 °C in a light-protected environment. When needed for experiments, the stock solutions were diluted to the desired concentrations using deionized water and stored briefly at 4 °C in a light-protected environment.

### 2.3. Preparation of Adsorbent Material

Improvements were made to the preparation method from previous literature [25]. Activated CNTs were prepared using 1.0 g of CNTs refluxed in nitric acid at 90 °C for 12 h, followed by rinsing with deionized water and drying. A mixture of activated CNTs (0.50 g), sodium acetate (3.60 g), FeCl_3_·6H_2_O (3.06 g), and ethylene glycol (80.0 mL) was heated in a constant temperature oven at 200 °C for 12 h and then cooled to room temperature. The precipitate magnetic carbon nanotubes (MCNTs) were rinsed with deionized water and dried. A mass of 400 mg MCNTs was dispersed in a volume of 10.0 mL deionized water and 30.0 mL of isopropanol by ultrasound for 15 s. Then, an ammonia solution (20.0 mL) and tetramethyl orthosilicate (10.0 mL) were added. The solution was stirred at 25 °C for 12 h, washed with a magnet and deionized water, and dried. Modified MCNTs were obtained and dissolved in 40.0 mL of deionized water in a flask. Then, 3-aminophenylboronic acid (58 mg) and 2-deoxy-D-ribose (26 mg) were added to the flask and stirred at 25 °C for 1 h. Subsequently, 215 mg of ammonium persulfate and 200 mg of N,N’-methylenebisacrylamide were dissolved in water (20.0 mL), added to the flask, and heated and stirred at 80 °C for 12 h. The product was separated with a magnet, washed several times with deionized water by shaking, and dried at 45 °C to obtain MMIPs. The template removal was monitored by the HPLC-DAD system after derivatization of 2-deoxy-D-ribose. Under the same conditions, magnetic carbon nanotube non-molecular template molecularly imprinted polymers (MNIPs) were synthesized using the same method as MMIPs, but without the addition of 2-deoxy-D-ribose.

### 2.4. Characterization of Adsorbent Materials

MMIPs were characterized for functional groups using Fourier transform infrared spectroscopy (FTIR, Thermo Scientific Nicolet iS20, Norristown, PA, USA). Magnetic properties were analyzed using vibrating sample magnetometry (VSM, LakeShore7404, Westerville, OH, USA), morphology and internal encapsulation were evaluated and analyzed using scanning electron microscopy (SEM, ZEISS GeminiSEM 300, Oberkochen, Germany) and transmission electron microscopy (TEM, JEOL JEM 2100, Tokyo, Japan), and the component analysis of MMIPs and MCNTs was performed using thermogravimetric analysis (TG, TA TGA 550, New Castle, DE, USA).

### 2.5. Derivatization and Instrument Method Development

**Figure 1:**
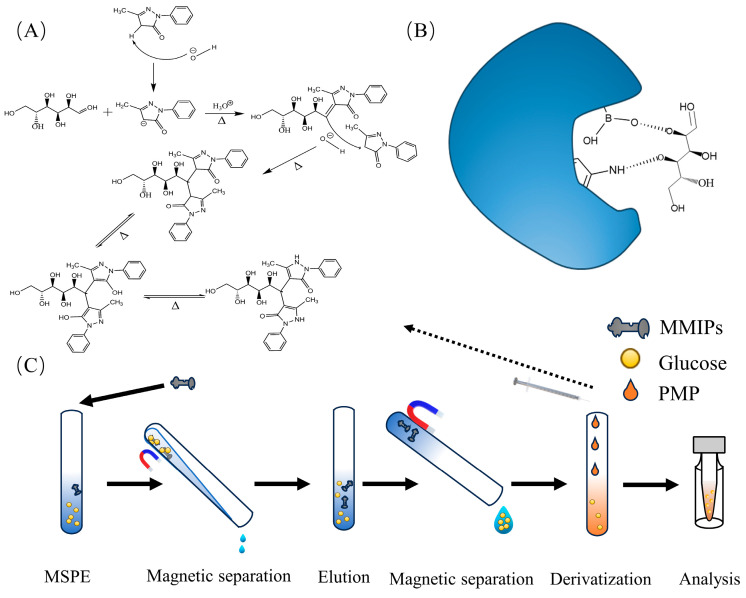
(**A**) Reaction of glucose derivatization; (**B**) mechanism of sorbent adsorption; (**C**) process of MSPE experiments.

The derivatization mechanism is illustrated in Figure 1A. An amount of 2 mL standard sugar solution was taken, and 1 mL of PMP–methanol solution (87 mg/mL) and 200 μL of ammonia were added. The mixture was incubated in a water bath shaker (90 °C, 1 h), cooled to room temperature after completion of the reaction, and diluted to 3 mL with deionized water. Sugars in the fish serum were separated using a C18 chromatographic column with mobile phase A consisting of 0.1% formic acid in deionized water and mobile phase B consisting of ACN. The gradient program was set as follows: 0–1 min: 20% B; 1–2 min: 20–95% B; 2.0–3.6 min: 95% B; 3.60–3.61 min: 95–20% B; 3.61–5.00 min: 20% B. Column temperature: 30 °C. Flow rate: 0.350 mL/min. The column eluate was monitored using an Agilent instrument (Santa Clara, CA, USA) equipped with a positive mode electrospray ionization interface (ESI+). The parameters were set as follows: gas temp., 250 °C; gas flow, 7 L/min; nebulizer 35 psi; sheath gas heater, 350 °C; sheath gas flow, 11 psi; capillary 3500 V. Multiple reaction monitoring (MRM) mode was used for target compounds with the following MRM transitions: glucose: 511.5–217.2 m/z, CE = 35 eV, fragmentor = 135 eV; 511.5–175.1* m/z, CE = 30 eV, fragmentor = 135 eV (*quantification ion pair).

### 2.6. Experimental Binding Assays

The adsorption mode is illustrated in Figure 1B. In the kinetic adsorption experiment, MMIPs were dispersed in a solution containing 400 μg/mL glucose. The mixture was mechanically shaken at room temperature for different time intervals (5, 10, 15, 20, 30, 40, 45, 50, 70, and 80 min). Subsequently, the adsorbent material was separated using a magnet, and the supernatant was collected. Glucose in the supernatant was derivatized and analyzed using HPLC-MS/MS for Glu-PMP.

In the static equilibrium adsorption experiment, a screw-cap centrifuge tube was used as an intermittent reaction system. MMIPs and MNIPs were dispersed in solutions of different concentrations (400, 500, 600, 800, 2000, 4000, 6000, and 8000 μg/mL). After adsorption, the supernatant was collected for derivatization, and the glucose derivatization product was determined using HPLC-MS/MS to confirm the adsorption capacity of glucose onto MMIPs and MNIPs.

Selective adsorption experiments were conducted by incubating standard mixed solutions of glucose and sucrose at a concentration of 1000 μg/mL separately with MMIPs and MNIPs for 20 min, followed by extraction, as described in the previous adsorption experiments.

### 2.7. Application of Fish Serum Samples

Firstly, fish blood was obtained and collected in sterile centrifuge tubes. The collected blood samples were placed in sterile centrifuge tubes at 4 °C until blood layering occurred. Subsequently, the tubes were centrifuged at 4 °C for 10 min, and the supernatant was collected. A volume of 100 μL of serum was taken and diluted to 2 mL with deionized water. The subsequent procedure was conducted as shown in Figure 1C. Specifically, a mass of 15 mg of MMIPs was added to the diluted sample and incubated for 20 min at 120 rpm. After MSPE, MMIPs were separated using a magnet, followed by ultrasonic elution with deionized water as the solvent. Subsequently, derivatization was performed, and the volume was adjusted to 2 mL with deionized water. Finally, qualitative and quantitative analyses of the derivatization product were conducted using HPLC-MS/MS.

## 3. Results and Discussion

### 3.1. Mechanism of Polymerization Reaction

The principles of MIPs technology mainly involve three stages: the first stage involves the formation of host–guest complexes between the functional monomer and the template molecule in the reaction medium, which is achieved through covalent or non-covalent interactions between functional groups. The second stage involves the addition of a cross-linking agent, which, catalyzed by the initiator, undergoes a polymerization reaction in the solution, forming polymer chains that encapsulate the functional monomer–template complex. The third stage involves the desorption of the template molecules encapsulated in the polymer, which can be achieved by either physical or chemical methods. After desorption, the polymer matrix retains its spatial geometries, the size and shape of which match the template molecule, and the pore contains functional groups complementary to the functional groups of the template molecule, imparting selective recognition characteristics to the cavity [26]. The adsorption mechanism of MMIPs is illustrated in Figure 1B. The synthesis of MMIPs begins with the pre-polymerization of the functional monomer 3-aminophenylboronic acid and the template molecule 2-deoxy-D-ribose to form a composite. Subsequently, cross-linker N,N’-methylenebisacrylamide, MCNTs, and initiator ammonium persulfate are introduced and cross-linked under 80 °C water bath conditions to form aromatic-containing MMIPs. Following this, the template molecules are removed through oscillation elution, resulting in the formation of amino-functionalized spatial pores. The hydroxyl groups present in the proximity of boron and amino groups are on the pore surface, where they can form chemical bonds with the hydroxyl groups in the glucose molecules. These amino groups and hydroxyl groups on the pore surface originate from the 3-aminophenylboronic acid. Previous research [27,28] has demonstrated that the functional monomer 3-aminophenylboronic acid exhibits specific recognition properties for glucose molecules. These conditions enable the selective adsorption of glucose molecules by MMIPs materials.

### 3.2. Characterization of Adsorption Materials

**Figure 2:**
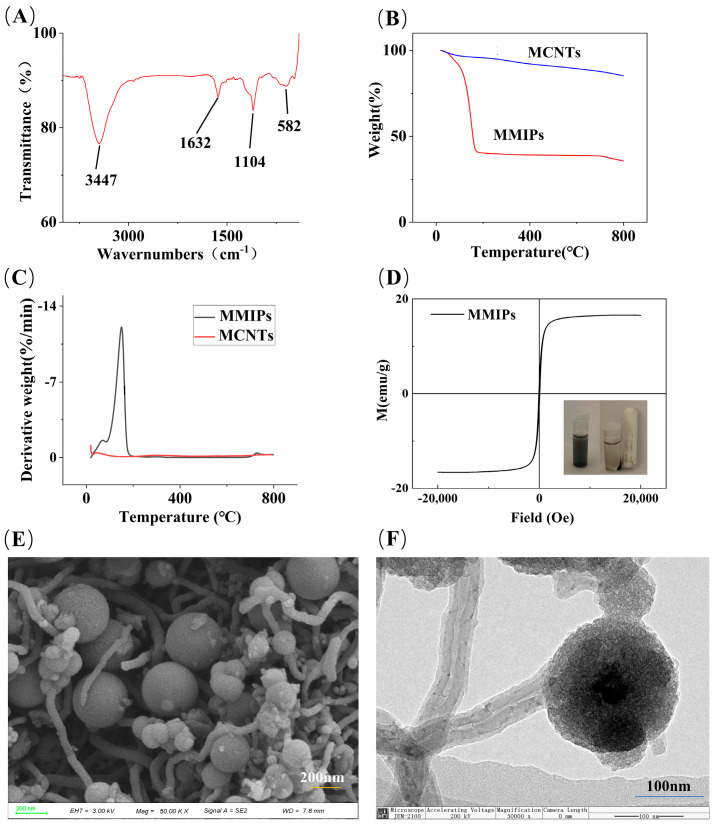
(**A**) FTIR spectrum of MMIPs; (**B**) TGA curves of MMIPs and MCNTs; (**C**) plot of TGA derivative weight data; (**D**) VSM plot of MMIPs; (**E**) SEM image; and (**F**) TEM image of MMIPs.

The FTIR spectrum of MMIPs is shown in Figure 2A. The peak at 3447 cm^−1^ is attributed to the stretching vibration of the N-H bond of SiO_2_@NH_2_ and the O-H bond in the adsorption material during the treatment process [29]. The absorption peak at 1104 cm^−1^ corresponds to the antisymmetric stretching vibration of the Si-O-Si bonds [30]. The peak at 582 cm^−1^ corresponds to the absorption peak of the Fe-O bond in the magnet [31]. The peak at 1632 cm^−1^ corresponds to the carbon–carbon double bond (C=C) and the skeleton vibration of the benzene ring [32]. The FTIR spectrum results indicate the successful synthesis of the MMIP materials.

The TGA curves and their derivative weight data for MCNTs and MMIPs are shown in Figure 2B and Figure 2C, respectively. MCNTs lost 14% of their mass in the range of 0–800 °C, mainly due to the loss of water molecules and other impurities (the remaining impurities in the process of CNTs and MCNTs synthesis or purification include residual metal catalysts, oxidized metal particle impurities, amorphous carbon, residual reagents, and nanocarbon microspheres, among others) [23]. The loss observed between 90–100 °C in MMIPs is attributed to residual water molecules and other impurities, while the 53% mass loss occurring between 100–200 °C is caused by the thermal cleavage of the functional monomer 3-aminophenylboronic acid and the cross-linking agent N,N′-methylenebisacrylamide. The mass loss observed after reaching 600 °C is mainly due to the loss of bound water in SiO_2_ [33].

The hysteresis curve, as shown in Figure 2D, indicates that MMIPs exhibit typical superparamagnetism, with a saturation magnetization intensity of 16.60 emu/g. According to previous experimental data from the research group, the magnetic intensity of magnetic carbon nanotubes without encapsulation layers is 25.69 emu/g [34]. As illustrated in the schematic diagram in Figure 2D, although the magnetic intensity of MCNTs decreases after encapsulation and modification of the outer layer, it does not affect the magnetic separation of materials in the MSPE process, and MMIPs can still be magnetically separated from complex matrices.

SEM and TEM were used to evaluate the morphology of MMIPs (Figure 2E,F). Figure 2E shows that MMIPs are composed of tubular and spherical structures adhering to each other. Figure 2F illustrates the combined state of tubular and spherical structures, with a diameter of 200 nm for the spherical structures. A thin imprint layer is observed on the entire surface of the spherical structure and the tubular structure, and there is a silicon layer encapsulating the outer layer of the tubular structure and the inner CNTs. These results confirm the successful synthesis of MMIPs.

### 3.3. Adsorption Performance of Adsorption Materials

#### 3.3.1. Adsorption Isotherms

**Figure 3:**
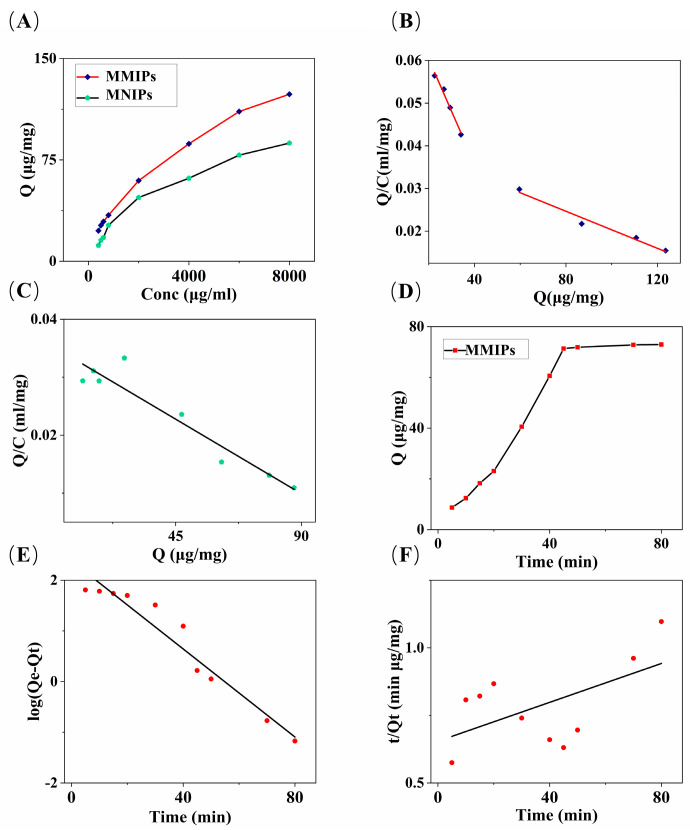
(**A**) Adsorption isotherms of MMIPs and MNIPs; (**B**) Scatchard analysis of MMIPs; (**C**) Scatchard analysis of MNIPs; (**D**) the effect of the contact time on the absorption capacity of MMIPs; (**E**) the pseudo-first-order kinetics; and (**F**) the pseudo-second-order kinetics for MMIPs.

The adsorption mode and the distribution of binding sites between glucose and MMIPs were evaluated through adsorption experiments. In the adsorption experiment, a certain mass of MMIPs was incubated with different concentrations of glucose until equilibrium was reached. The formula for calculating the equilibrium adsorption capacity of MMIPs is

(1) $$Q=\frac{\left(C_{i}-C_{e}\right)\times V}{m}$$

where *Q* (μg/mg) represents the equilibrium adsorption capacity of MMIPs for glucose, *C_i_* (μg/mL) is the initial solution concentration, *C_e_* (μg/mL) is the final solution equilibrium concentration, *V* (mL) is the volume of the measured solution, and *m* (mg) is the mass of MMIPs. The obtained equilibrium adsorption capacity data were used to plot the adsorption isotherm (Figure 3A). According to the adsorption data, the adsorption capacity of the MMIPs at each concentration point was always higher than that of the MNIPs. 

The Scatchard equation was used to further process the adsorption data of MMIPs (Figure 3B) and MNIPs (Figure 3C) [35]:

(2) $$\frac{Q}{C}=\frac{Q_{max}-Q}{K_{d}}$$

where *Q* (μg/mg) represents the equilibrium adsorption capacity, *C* (μg/mL) represents the equilibrium concentration of glucose, *Q_max_* (μg/mg) is the maximum adsorption capacity, and *K_d_* (μg/mL) is the equilibrium dissociation constant of the glucose binding sites. The *Q_max_* and *K_d_* values were calculated based on the intercept and slope of the linear plot of *Q/C* versus *Q*. The obtained *Q* values were the following: *Q_MMIPS_max__*_1_ = 69.24 μg/mg, *Q_MMIPs_max__*_2_ = 193.62 μg/mg, *Q_MMIPs_max_* = *Q_MMIPs_max__*_1_ + *Q_MMIPs_max__*_2_, and *Q_MMIPs_max_* = 262.86 μg/mg; *Q_MNIPs_max_* = 124.69 μg/mg. Based on these data, it can be observed that the first four concentration points can be fitted to one curve, and the remaining four points can be fitted to another curve, suggesting the existence of two adsorption binding modes between the MMIPs and the glucose molecules. 

The adsorption capacity of MMIPs obtained in our study exceeds the maximum adsorption capacity reported in the literature for adsorbent materials based on Fe_3_O_4_@SiO_2_ carriers [30,36]. The reason for this outcome lies in the introduction of CNTs into our synthesized adsorbent material solely as carriers. CNTs not only enhance the stability of the material but also improve the dispersibility of the adsorbent [37]. In contrast, when preparing magnetic adsorbent materials using Fe_3_O_4_@SiO_2_ as the carrier for adsorbing the glucose molecules, the maximum adsorption capacity obtained was 9.112 μg/mg [30], which is lower than that observed in our experimental results (*Q_max_* = 262.86 μg/mg). This discrepancy may be attributed to the heterogeneous nature affecting the adsorption capacity of Fe_3_O_4_@SiO_2_ microspheres [38]. In contrast, our material, with the incorporation of MCNTs as the adsorbent carrier, not only enhances dispersibility but also increases magnetic properties. As illustrated in Figure 2E,F, due to the high surface area of carbon nanotubes, the addition of MCNTs allows the microspheres to separate from each other, increasing the contact area between the microspheres and the target material, thereby enhancing the adsorption capacity of the material [34] and increasing the maximum adsorption capacity. 

At low concentrations, MMIPs adsorb glucose molecules through specific binding sites, while at high concentrations, all binding sites are occupied by glucose molecules, and the remaining glucose molecules are physically adsorbed by the adsorbent. After the specific adsorption sites are saturated, substances are adsorbed to non-specific binding sites. MNIPs, on the other hand, cannot undergo specific adsorption due to the lack of specific binding sites, so only one curve can be fitted according to the experimental data, which may represent the non-specific adsorption of MNIPs.

#### 3.3.2. Adsorption Kinetics

The adsorption time curve is shown in Figure 3D. As the oscillation time increases, the equilibrium adsorption capacity of MMIPs gradually increases. After 45 min of adsorption time, the equilibrium adsorption capacity almost reaches saturation, and it reaches the maximum at 80 min. The pseudo-first-order and pseudo-second-order rate equations [39] were used to analyze the kinetic data, as shown in Figure 3E,F. The pseudo-first-order and pseudo-second-order rate equations are, respectively,

(3) $$\log\left(q_{e1}-q_{t}\right)=\log q_{e1}-k_{1}t$$

(4) $$\frac{t}{q_{t}}=\frac{1}{k_{2}q_{e2}^{2}}+\frac{t}{q_{e2}}$$

the pseudo-first-order model yielded *k*_1_ = 0.04343 min^−1^, *q_e_*_1_ = 240.59 mg/g, and R^2^ = 0.942. The pseudo-second-order model yielded *k*_2_ = 0.00002 g mg^−1^ min^−1^, *q_e_*_2_ = 278.03 mg/g, and R^2^ = 0.323. The pseudo-first-order kinetic model primarily indicates that the adsorption process is controlled by diffusion, and it is a physical adsorption process [37]. Meanwhile, the pseudo-second-order kinetic model assumes that the adsorption process is chemically controlled through electron sharing or electron transfer between the adsorbent and the adsorbate [40]. The pseudo-first-order kinetic model obtained a better correlation coefficient, and its maximum adsorption capacity data were close to those obtained from the Scatchard equation analysis. According to the Scatchard equation analysis, the maximum adsorption capacity data were *Q_MMIPs_max__*_2_ = 193.62 μg/mg, which is greater than *Q_MMIPs_max__*_1_ = 69.24 μg/mg. This demonstrates that the adsorption process of glucose molecules by MMIPs is primarily dominated by the pseudo-first-order kinetic model, indicating that the entire process is achieved through the interaction between the adsorbate diffusion step and the adsorbent. The adsorption mode is predominantly physical adsorption. The reason for the predominance of physical adsorption in the adsorption mode may be that glucose molecules are small molecules, with a relatively simple structure, making them easily physically attachable to the high adsorption capacity of the MMIP adsorbent material using MCNTs as a carrier. Therefore, the adsorption kinetics of MMIPs on glucose are more in line with the pseudo-first-order kinetic model.

#### 3.3.3. Adsorption Selectivity

To determine the selective adsorption of the synthesized MMIPs on glucose, a comparative study with sucrose was conducted. The following formulas were used for calculation:

(5) $$∆=\frac{Q}{C}$$

(6) $$\theta =\frac{{∆}_{glucose}}{{∆}_{surcose}}$$

(7) $$\zeta =\frac{\theta_{MMIPs}}{\theta_{MNIPs}}$$

here, Δ represents the static distribution coefficient, *Q* represents the equilibrium adsorption capacity, *C* represents the concentration of the sugar standard solution, *θ* represents the selectivity coefficient of MMIPs or MNIPs, and *ζ* represents the ratio of selectivity coefficients. The calculated values were *θ_MMIPs_* = 14.56; *θ_MNIPs_* = 5.80; ζ = 2.51. Based on the obtained data, MMIPs exhibit strong selectivity and imprinting effect. This result is related to the imprinting effect, the difference in molecular weight between glucose and sucrose molecules, and the interaction between the functional groups of the target molecule and the imprint cavity.

### 3.4. Sample Pretreatment Condition Optimization

To further optimize the experimental conditions and improve the recovery rate of the adsorbent for glucose, an analysis of fish serum samples was conducted. The parameters affecting the MSPE operation were investigated. The optimization was performed on the dosage of MMIPs during the adsorption process, oscillation time, oscillation rate, and the elution solution during the elution process. Finally, experimental studies were conducted on the repeatability of material for reuse.

#### 3.4.1. Adsorbent Mass

**Figure 4:**
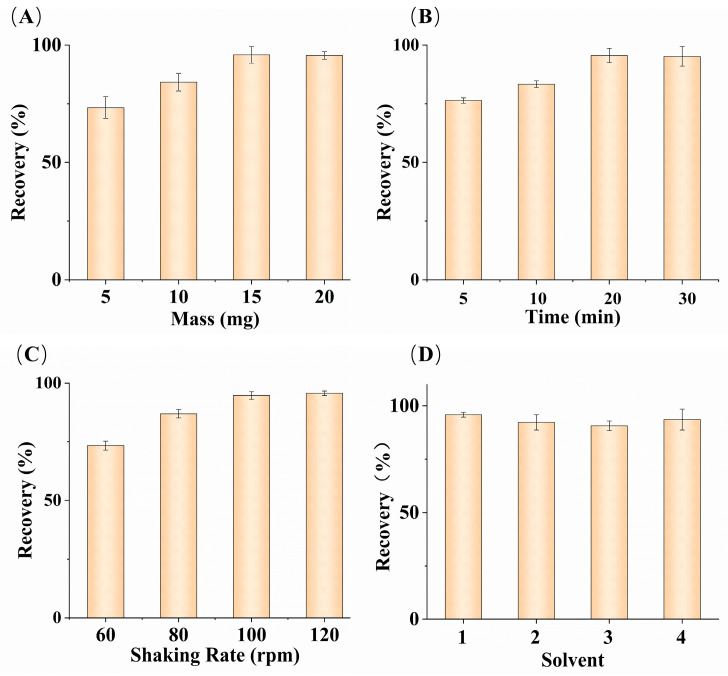
Effects of MMIPs mass (**A**); oscillation time (**B**); oscillation rate (**C**); different elution solutions (**D**). ((**D**), solvent (1) deionized water; (2) 20% ACN–water solution; (3) 20% methanol–water solution; (4) mixture of 20% ACN and 80% ethanol) (n = 3).

During the adsorption process, the MMIPs were dispersed in fish serum samples with the aim of using the minimum amount of MMIPs to achieve a satisfactory glucose recovery. Different masses of adsorbents (5–20 mg) were studied (Figure 4A). The increase in the recovery rate declined when the adsorbent mass reached 20 mg. Satisfactory recovery rates were obtained using 15 mg of MMIPs, and there was no significant change in the recovery rate with increasing mass. Therefore, the subsequent experiments used 15 mg of MMIPs.

#### 3.4.2. Oscillation Time

Oscillation time is also an important factor during the adsorption process. Sufficient time is required to extract glucose from fish serum samples to achieve equilibrium and obtain satisfactory recovery rates. According to Figure 4B, with other conditions being equal, the recovery rate increased continuously with time in the range of 5–20 min. After 20 min, the increasing trend of the recovery rate gradually leveled off, and there was almost no change in the recovery rate between 20 and 30 min. Considering the overall situation, an oscillation time of 20 min was selected as the optimal condition.

#### 3.4.3. Oscillation Rate

Different oscillation rates were applied to fish serum samples to calculate the differences in glucose recovery rates under different oscillation adsorption rates and determine the optimal oscillation rate. The optimization results are shown in Figure 4C, where the recovery rate gradually increased within the range of 60–120 rpm. After 100 rpm, the rate of increase in the recovery rate declined. The glucose in fish serum is adsorbed by being fully exposed to MMIPs, and the higher the oscillation rate, the more frequent the contact between the adsorbent and glucose molecules. The highest recovery rate was achieved at 120 rpm, making this the optimal oscillation rate.

#### 3.4.4. Elution Solvent

Different solvents were used to elute the adsorbent, and the influence of different elution reagents on glucose recovery rate was calculated. As shown in Figure 4D, deionized water, a 20% acetonitrile–water solution, a 20% methanol–water solution, and a mixture of 20% acetonitrile and 80% ethanol were used as elution solvents for the elution experiment. The highest recovery rate was obtained with the deionized water, while the recovery rate data obtained from the 20% acetonitrile–water solution and the mixture of 20% acetonitrile and 80% ethanol were close. Different elution reagents caused different effects on the glucose recovery rate, primarily due to differences in the binding structure of glucose molecules in different elution solvents, as well as differences in the solubility of glucose in different elution reagents. Considering the environmental pollution from organic solvents, deionized water was the most suitable elution solvent for the adsorbent.

#### 3.4.5. Reusability

We conducted experiments on the reuse of the MMIP adsorbent material. The previously used 15 mg of MMIP material was reused multiple times using the same pretreatment method for the fish serum samples as before. The experimental conditions were the optimized conditions, and even after multiple reuses, the recovery rate remained above 70%.

### 3.5. Method Validation

The combined method of MSPE with HPLC-MS/MS was validated. The data are shown in Table 1. The quantitative calibration curve of glucose was obtained from the glucose standard solution calibration graph of the analyte. After the MSPE pretreatment, the linear range of glucose detection by HPLC-MS/MS was from 0.99 ng/mL to 10,000 ng/mL, with a correlation coefficient of 0.993, indicating a linear relationship. The LOD and LOQ were measured as the lowest injected concentrations in the samples, with the signal/noise ratios of 3 and 10, while the LOD and LOQ for glucose were 0.30 ng/mL and 0.99 ng/mL, respectively. The recovery rate of glucose ranged from 93.09% to 102.41%, with an intra-day relative standard deviation (RSD) of 0.5–1.1% and an inter-day RSD of 2.4%.

### 3.6. Method Comparison

The method was compared with other methods, and the comparison results are shown in Table 2. It can be seen that, due to the selectivity of MMIPs and the magnetic separation of the adsorbent from the sample matrix, this method can handle samples in batches in a relatively short time while ensuring selective enrichment of glucose in complex matrices. Compared to the methods reported in the literature for purifying glucose using MIPs, using 2-deoxy-D-ribose as a template molecule can avoid false positives in experimental results and reduce measurement errors during quantification. Selecting MCNTs as the carrier for the adsorbent can significantly increase the equilibrium adsorption capacity of the adsorbent. The equilibrium adsorption capacity of the MMIP material we prepared is more than four times that of its predecessor. Compared to the results reported in previous methods, the range of our relative standard deviation (RSD) is quite excellent, resulting in a higher sensitivity and lower LOD and LOQ. In addition, compared to the sensor detection method, we obtained 30–8000 times higher sensitivity. Therefore, we can analyze the glucose content using a small amount of sample, achieving trace sampling or even non-destructive sampling for the accurate determination of the glucose content. 

### 3.7. Application on Real Samples

**Figure 5:**
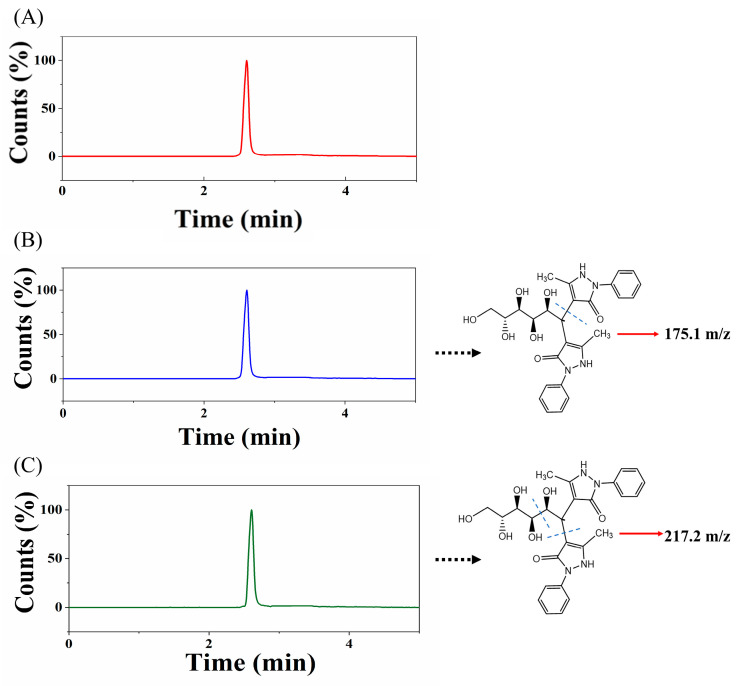
(**A**) Total ion chromatogram of fish serum glucose detection; (**B**) glucose derivative 511.5–175.1 m/z extracted ion chromatogram; (**C**) glucose derivative 511.5–217.2 m/z extracted ion chromatogram.

After optimizing the relevant conditions and parameters, the method was successfully applied to the analysis of fish serum samples for glucose. The total ion chromatogram (Figure 5A) and the extracted ion chromatograms (Figure 5B,C) were obtained. The glucose content in fish serum was determined to be 757.43 μg/mL. The experimental results obtained using the method specified in the national standard GB5009.8-2023 [46] (National Food Safety Standards—Determination of Fructose, Glucose, Sucrose, Maltose, and Lactose in Foods) yielded a glucose concentration of 695.65 μg/mL. The results obtained using a commercial assay kit were 704.40 μg/mL. The RSD was 5.1–6.0%, and the results obtained by both methods were similar to those obtained using our method. The glucose assay kit method requires at least 10 μL of serum sample, while our method only requires 0.2 nL for the qualitative and quantitative determination of glucose.

## 4. Conclusions

MMIPs for purifying glucose were prepared using MCNTs as carriers and 2-deoxy-D-ribose as a template molecule. The recognition characteristics and applications of MMIPs based on MSPE were evaluated. The obtained MMIPs exhibited a high adsorption capacity for glucose. After adsorption with MMIPs, followed by derivatization and analysis using HPLC-MS/MS in fish serum samples, this method demonstrated good enrichment performance, high selectivity, and sensitivity in glucose detection. Such novel imprinting materials may serve as powerful tools for the enrichment and purification of trace glucose from complex matrices such as saliva, serum, and urine, using low sample volumes. These advantages make surface-imprinted materials one of the most promising candidates for various applications, with extremely broad prospects.

## Figures and Tables

**Table 1:** Recovery and RSD toward the detection of glucose in spiked fish serum samples.

Concentration of Samples (μg/mL)	Spiked Concentration (μg/mL)	Detection of Average Results ± SD (μg/mL)	RSD (%)	Recovery (%)
750.00	10	757.76 ± 8.20	1.1	99.70
	100	870.51 ± 8.86	1.0	102.41
	1000	1629.16 ± 8.19	0.5	93.09

**Table 2:** Comparison of the developed method with other methods for the determination of glucose.

Matrix	Method of Purification	Determination	LOD	LOQ	Recovery (%)	RSD (%)	References
Mucilage of *Opuntia ficus indica* cladodes	Filtration–Purification	HPLC-MS/MS	500 ng/mL	5000 ng/mL	96.30–118.10	4.66	[41]
Various fruit	Glu–MIPs	MISPE-HPLC	2.0 × 10^6^ ng/g	6.6 × 10^6^ ng/g	93.95–109.12	0.89–4.80	[18]
Fruit, honey and jam	Filtration–Purification	HPLC-ELSD	160 ng/mL	490 ng/mL	102.28	0.27	[3]
Jujube	Filtration–Purification–Derivatization	HPLC-DAD	2 × 10^7^ ng/g	4 × 10^7^ ng/g	105.07	3.35	[23]
Human sweat	Dilution	colorimetric sensor (paper-based)	2428 ng/mL	-	76.72–108.87	0.7–3.4	[42]
Human serum	Dilution	glucose sensor (electrochemical enzyme-free)	187.2 ng/mL	-	99.38–102.16	0.78–1.77	[43]
Sugarcane juice	Dilution	glucose sensor (Copper/Cobalt bilayered nanowires)	9.8 ng/mL	29.6 ng/mL	-	less than 3.3	[44]
Tear fluid	Filtration–Purification	glucose sensor (inkjet-printed flexible non-enzymatic)	538.2 ng/mL	-	92.8	5.9–9.8	[45]
Serum of fish	MMIPs	HPLC-MS/MS	0.30 ng/mL	0.99 ng/mL	93.09–102.41	0.5–1.1	This method

## Data Availability

Data are contained within the article.
